# Art and science: impact of semioccluded vocal tract exercises and choral singing on quality of life in subjects with congenital GH deficiency

**DOI:** 10.20945/2359-3997000000449

**Published:** 2022-03-23

**Authors:** Bruna M. R. de Andrade, Eugenia H. O. Valença, Roberto Salvatori, Luiz A. Oliveira, Anita H. O. Souza, Alaíde H. A. Oliveira, Mario C.P. Oliveira, Enaldo V. Melo, Susana de Carvalho, Neuza J Sales, Gisane C. Monteiro, José Marcel de Lima, Marcos Felipe Harder Annunziato, Guilherme Daniel Breternitz Mannis, Lucas E. de A. Souza, Yasmin D. Goes, Thayza S. Carvalho, Celiane de Farias, Michela P. dos Santos, Gabriela P. F. Cardoso, Carla S. Pereira Sousa, Julia Rodrigues Santana, Ester Almeida Sales, Jeferson Sampaio d’Avila, Manuel H. Aguiar-Oliveira

**Affiliations:** 1 Universidade Federal de Sergipe Departamento de Fonoaudiologia Aracaju SE Brasil Departamento de Fonoaudiologia, Programa de Pós-graduação em Ciências da Saúde, Universidade Federal de Sergipe, Aracaju, SE, Brasil; 2 The Johns Hopkins University School of Medicine Baltimore (Maryland) Department of Medicine Division of Endocrinology United States Division of Endocrinology, Department of Medicine, The Johns Hopkins University School of Medicine Baltimore (Maryland), United States; 3 Universidade Federal de Sergipe Divisão de Odontologia Aracaju SE Brasil Divisão de Odontologia, Universidade Federal de Sergipe, Aracaju, SE, Brasil; 4 Universidade Federal de Sergipe Divisão de Endocrinologia Aracaju SE Brasil Divisão de Endocrinologia, Programa de Pós-graduação em Ciências da Saúde, Universidade Federal de Sergipe, Aracaju, SE, Brasil; 5 Universidade Federal de Sergipe Departamento de Comunicação Social Aracaju SE Brasil Departamento de Comunicação Social, Universidade Federal de Sergipe, Aracaju, SE, Brasil; 6 Universidade Federal de Sergipe Aracaju SE Brasil Fonoaudiologia, Programa de Pós-graduação em Ciências da Saúde, Universidade Federal de Sergipe, Aracaju, SE, Brasil; 7 Conservatório de Música de Sergipe Aracaju SE Brasil Conservatório de Música de Sergipe, Aracaju, SE, Brasil; 8 Orquestra Sinfônica de Sergipe Aracaju SE Brasil Orquestra Sinfônica de Sergipe, Aracaju, SE, Brasil; 9 Universidade Federal de Sergipe Divisão de Otorrinolaringologia Aracaju SE Brasil Divisão de Otorrinolaringologia, Programa de Pós-graduação em Ciências da Saúde, Universidade Federal de Sergipe, Aracaju, SE, Brasil

**Keywords:** Growth Hormone, voice, singing, quality of life, auditory-perception analysis

## Abstract

**Objectives:**

Currently, not much is known about the interactions between voice and growth hormone (GH). We have described large kindred with isolated GH deficiency (IGHD) due to a GHRH receptor mutation, resulting in severe short stature and high-pitched voice. These IGHD individuals have little interest in GH treatment, as they consider themselves “short long-lived people”, rather than patients. Interestingly, they report normal general quality of life, but they rate their Voice-Related Quality of Life (V-RQOL) as low. Here, we assessed the social and auditory-perceptual impacts of artistic-intervention voice therapy with semioccluded vocal tract exercises (SOVTE) and choral singing, on their voices.

**Material and methods:**

Seventeen GH-naïve adult IGHD individuals were enrolled in a single-arm interventional pre-post study with 13 weekly sessions of choir singing over 90 days. Outcome measures were V-RQOL scores, self-assessment of voice, and auditory-perceptual analysis (GRBAS scale, G: grade of the severity of dysphonia; R: roughness; B: breathiness; A: asthenia; and S: strain).

**Results:**

Marked improvements in total (p = 0.0001), physical (p = 0.0002), and socioemotional (p = 0.0001) V-RQOL scores and in self-assessment of voice (p = 0.004) were found. The general grades of vocal deviation (p = 0.0001), roughness (p = 0.0001), breathiness (p = 0.0001) and strain (p = 0.0001) exhibited accentuated reductions.

**Conclusions:**

Voice therapy with semioccluded vocal tract exercises and choral training improved social impact and perceptual voice assessments in IGHD subjects and markedly improved their voice-related quality of life. This is particularly important in a setting where GH replacement therapy is not widely accepted.

## INTRODUCTION

Art can reveal secrets unknown to science. The Brazilian physician and researcher Carlos Chagas, nominated four times for the Nobel Prize in Medicine for describing American trypanosomiasis, wrote: “It won’t be long before we pass on great and beautiful science, which makes art in defense of life”. Art can have an experimental method, and science can contribute to rethinking artistic processes. Experiments are important points for successful art-science collaboration ( 1 ). Indeed, painting, theater, music, dance, and singing, among other artistic activities, are increasingly used to treat various illnesses and deserve scientific assessment to prove their effectiveness. Singing, the vocal production of musical tones, is a way of expressing feelings and emotions. As it is ancient and universal, singing has its origins prior to the development of spoken language and deserves attention for being integral to human social life ( 2 ). For over 25 years, we have been studying subjects with isolated congenital growth hormone (GH) deficiency (IGHD) residing in Itabaianinha in northeastern Brazil and discovered that their IGHD is due to an autosomal recessive *null* mutation (c.57+1G>A) in the GH-releasing hormone receptor (GHRHR) gene ( 3 – 5 ). We followed Dr. Chagas’s wise prediction by assessing the impact of an artistic activity, choral singing, on the physical and social-emotional aspects of the singer’s voice. Stature and voice are fundamental features of self-confidence and social acceptance, and both are affected by GHD.

Voice is a genetic trait ( 6 ) but simultaneously a skill that can be influenced by environmental and/or training conditions. It is one of the most striking characteristics of each person’s uniqueness, and in addition to its physical properties, it has perceptual and social aspects that are extremely important for environmental adaptation. Voice can be thoroughly evaluated by multidimensional voice assessment. This includes objective acoustic analysis and subjective measures, namely, Voice-Related Quality of Life (V-RQOL) scores and auditory-perceptual analysis. The VRQOL is a 10-item instrument that was designed and validated as a self-administered instrument for adult populations with voice disorders to measure both the social-emotional and physical-functional aspects of voice problems ( 7 , 8 ). Auditory-perceptual analysis is the gold standard for characterizing vocal problems. This stems mainly from the fact that the quality of the voice is perceptual in nature and that the perceptual characteristics of the voice have a greater intuitive meaning than many instrumental measures.

Itabaianinha IGHD subjects exhibit very low serum levels of GH and its principal effector IGF-1 throughout their life ( 9 ). Despite this, they exhibit an apparent benign phenotype, being quite active with satisfactory muscular function ( 10 ), and autonomy even at advanced ages ( 4 ). The principal physical findings of these IGHD individuals are severe short stature, central obesity (with reduced muscle mass), wrinkled skin, with a “doll” face or cherubim angel face (due to the disproportion between the calvarium and the face), and a high-pitched voice ( 4 , 5 , 11 ), with increased values of fundamental frequency ( *f*_0_) ( 12 , 13 ) and of most formant frequencies ( 14 ). Their voices have higher values for roughness, breathiness and strain associated with a high frequency of laryngeal constriction ( 12 ). Interestingly, these individuals seem to cope better with their short stature than with their voice, since they rate their overall quality of life as excellent ( 15 ) but rate their V-RQOL as low ( 13 ). To examine this dissociation, until now, we had focused on physical aspects, such as reduced head circumference ( 13 ) and facial depth, cephalometric changes, and reduction of most hard ( 16 ) and soft structures of the vocal tract, except the pharyngeal airway width ( 17 ). However, the impacts of these changes on the social aspects of the voice are not presently known.

GH replacement therapy (GHRT) administered during childhood increases the stature of these IGHD individuals; however, most of them do not have interest in GHRT. Unlike what is seen in the Western world, after the initial interest in treatment, most children and their parents have little interest in maintaining a long-term and daily injectable GHRT, as they do not consider short stature a disadvantage. Consequently, we sought an intervention that could improve their voice without using GHRT. One of these approaches is choir singing, which leads to important improvement in vocal skills and acoustic variables in GH-sufficient children and adults ( 18 – 20 ); however, the role of choir singing in the voice of IGHD individuals is unknown.

We have previously shown that choir singing, coupled with voice therapy and semioccluded vocal tract exercises (SOVTE), over a 30-day period, has positive effects in the acoustic analysis of the voice of IGHD adults ( 19 ). Here, we report the effects of an art-science interaction study to verify whether these acoustic changes translate into social and auditory-perceptual effects in their voice, thus improving their quality of life related to voice.

## MATERIAL AND METHODS

A single-arm interventional pre-post study in which each IGHD subject acted as their own control ( 21 ) was performed using a multidimensional voice assessment.

The individuals were invited to participate in the vocal intervention through advertisements placed at the dwarfs’ association facilities located in Itabaianinha County, through phone calls and personal invitations at home and in regular meetings between the dwarf’s group and the research team. Most individuals were illiterate or had only a basic education. The most frequent professions were agricultural workers, commerce workers, and housewives. Only one female teacher had undergone speech therapy a few years earlier. The IGHD status was previously confirmed in this cohort based on abnormal GH responsiveness (with peak values less than 1 ng/mL) to clonidine, GHRH, and insulin-induced hypoglycemia associated with very low, often undetectable, IGF-1 levels ( 3 , 9 ).

The Research Ethics Committee of the Federal University of Sergipe (CEP/UFS) approved the protocol with CAAE reference number 74171317.8.0000.5546. Informed consent was obtained from all participants.

The inclusion criteria were Brazilian Portuguese language native speakers and homozygosity for the GHRHR gene c.57+1G>A mutation ( 15 ). The exclusion criteria were age under 19 years, previous GH treatment, presence of dysmorphic syndromes, speech cognitive impairments, and laryngeal diseases ( 12 ). Seventeen IGHD subjects, of which 9 were women, were enrolled with a mean (standard deviation) age of 48.6 years (15.8 years), with a range of 22 to 79 years, and height of 127.5 (9.9), with a range of 108 to 137 cm. As we assessed the effects of pre- and post-vocal intervention in the same subject, we used the data of pooled genders.

### Vocal Intervention: voice therapy with SOVTE and choral singing

The vocal intervention consisted of 13 combined weekly sessions of choir training (60 min) and voice therapy with SOVTE carried out for 90 days between September and December of 2018. The same speech therapist with experience in the rehabilitation of voice disorders performed all sessions. The individuals were instructed to perform the SOVTE for 10 minutes, with a silicone tube 35 cm long and 0.9 cm in diameter submerged for 2 cm in a 500-mL bottle of mineral water. Two series of 5 minutes each included the sequence of three repetitions of each emission of the vowel/u/: ( 1 ) short and sustained sounds; ( 2 ) ascending (bass-acute) and descending (acute-bass) glissandos; and ( 3 ) singing the song “happy birthday” ( 22 ). In the first series, the silicone tube was submerged for 2 cm, and in the second series, it was immersed 15 cm deep in water.

Thirteen choral rehearsals were conducted and taught by a qualified singing teacher with over 15 years of experience in the area. The classification of the voices for the division of the parts of the choir was determined through an auditory-perceptual evaluation of vocalizations of the vowel/ɔ/. By considering the prepubertal vocal pattern of these individuals with IGHD, choir training started with the second voice model, part of the low voice choir, followed by the high first voice ( 13 , 19 ).

### Vocal sample recording

Two steps were used for voice recording: first, 3 seconds of sustained emission of the vowel/ε/, second, continuous speech in counting numbers from 1 to 10, using a comfortable conversational pitch and loudness levels in a quiet room with < 40 dB of noise. The audio capture of the voices was performed with a unidirectional type, TSI brand model PRO BR-SW microphone, kept at a fixed distance of 5 cm from the mouth, coupled with a Shure® audio converter and amplifier, connected to an HP computer model G42-330BR notebook PC. V-RQOL scores and vocal self-assessment: The instrument to measure V-RQOL scores was a validated Brazilian Portuguese version ( 23 ). Two domains were recognized in the questionnaire, the total score results from the sum of 10 items, six from the physical and four from the socioemotional domains. The V-RQOL scores ranged from 0 to 100, with the highest scores indicating a high V-RQOL. The self-assessment of voice was rated based on a five-point categorical scale as follows: 1: excellent; 2: very good; 3: good; 4: fair or 5: poor ( 13 ). The V-RQOL scores and the self-assessment of voice were carried out by the members of the Laboratory of Voice, Speech and Fluency (LVFF) of the Department of Speech Therapy of the Federal University of Sergipe.

For auditory-perceptual analysis, the GRBAS scale was used ( 12 , 24 ), in which two tasks were evaluated: sustained/ε// vowel and counting number. The five-parameter grade of the general vocal deviation (G), roughness (R), breathiness (B), asthenia (A), and strain (S) were scored using four points: (0: absent, 1: mild, 2: moderate, and 3: severe). The judges were three speech therapists with more than five years of experience; the recorded audio samples were perceptually assessed in a blinded fashion. A high consistency indicated that the grade of vocal deviation was present in both tasks and for all judges. The judges of the auditory-perceptual analysis GRBAS scale were not involved in therapy with SOVTE and choir training.

### Statistics

The comparison of GRBAS scale grade, V-RQOL scores and self-assessment of voice was performed on the first pre-vocal intervention day and on the last post-vocal intervention day. The Shapiro-Wilk test was used to verify the normality of the distribution of V-RQOL scores. They were expressed as the mean (standard deviation) if they had a normal distribution and as the median (interquartile distance) if they had a nonnormal distribution.

For pre- and post-vocal intervention comparison of continuous variables, the T test for paired data was used, according to the assumption of normality, and the Wilcoxon test was used for nonnormal distribution. Categorical variables (GRBAS scale and self-assessment of voice) are expressed as numbers and percentages and were compared using the Wilcoxon test.

We also calculated the test score for observer reliability, the Cronbach’s alpha coefficient. A Cronbach’s alpha of 0.70 and above was good, 0.80 and above was better and 0.90 and above was the best. To compare vocal self-perception, the McNemar test was used, considering the significance of a p level less than 0.05 and power of 0.8. Statistical analysis was performed using the Statistical Package for the Social Sciences (SPSS) version 17 (PC Inc. Program, Chicago, IL).

## RESULTS

There was an accentuated increase in V-RQOL scores: total from 75.1 (9.5) to 96.2 (4.1), p = 0.0001; physical from 67.9 (12.2) to 94.1 (16.1), p = 0.0002; and socioemotional from 85.7 (11.4) to 99.6 (1.5), p = 0.0001. Table 1 shows the 95% confidence of these continuous variables.

**Table 1:** 95% confidence interval (95% CI) of the comparison between the moment’s pre- and postintervention and voice-related quality of life (V-RQOL) scores in 17 IGHD subjects

Parameters	t	Mean difference	95% CI
Total V-RQOL	-9.44	-18.8	-23.01 to -14.7
Physical V-RQOL	-11.8	-24.7	-29.02 to -20.33
Socioemotional V-RQOL	-4.67	-11.9	-17.19 to -6.58

HNR: harmonic-to-noise ratio.

Table 2 shows the comparison of pre- and postintervention data of the self-assessment of voice. Preintervention, 6 IGHD individuals rated their voice as very good, and 11 rated it as fair. Postintervention, 15 IGHD subjects rated their voice as very good, and 2 rated it as fair. Accordingly, the self-assessment of voice of the entire IGHD group improved significantly (p = 0.004).

**Table 2:** Comparison of pre-intervention and post-vocal intervention data of the self-assessment of voice in 17 IGHD subjects using the McNemar test

Variable	Preintervention		Post-intervention		p
	n	%	n	%	
1 = Excellent	6	35.3	15	88.2	0.004
2 = Very good					
3 = Good					
4 = Fair	11	64.7	2	11.8	
5 = Poor					

Table 3 shows the comparison of pre- and postintervention auditory-perceptual data of the GRBAS scale. GRBAS showed a marked reduction (or absence) in the grade of the general vocal deviation or in the five parameters. For general vocal deviation and preintervention, all 17 individuals had any grade of general vocal deviation (10 moderate and seven severe); postintervention, nine had an absent grade, and eight exhibited a mild grade of general vocal deviation.

**Table 3:** Comparison of preintervention and postintervention data of the auditory-perceptual analysis (GRBAS scale) in 17 IGHD subjects

Variables	n	Preintervention		Post-intervention		p
		n	%	n	%	
General Grade of vocal deviation	0	0	0	9	52.9	0.0001
	1	0	0	8	47.1	
	2	10	58.8	0	0	
	3	7	41.2	0	0	
Roughness	0	0	0	13	76.5	0.0001
	1	4	23.5	4	23.5	
	2	8	47.1	0	0	
	3	5	29.4	0	0	
Breathiness	0	1	5.9	17	100.0	0.0001
	1	7	41.2	0	0	
	2	8	47.1	0	0	
	3	1	5.9	0	0	
Asthenia	0	10	58.8	17	100.0	0.015
	1	4	23.5	0	0	
	2	3	17.6	0	0	
	3	0	0	0	0	
Strain	0	0	0	11	64.7	0.0001
	1	2	11.8	6	35.3	
	2	10	58.8	0	0	
	3	5	29.4	0	0	

0 = normal, 1 = mild deviation, 2 = moderate deviation, 3 = severe deviation, using the Wilcoxon test.

Before intervention, all 17 IGHD individuals had some grade of deviation for roughness (four mild, eight moderate and five severe). For strain (two mild, 10 moderate, and five severe), after intervention, we observed a mild deviation (p = 0.0001) for roughness (four) and for strain (six). Before intervention, almost all subjects had some grade of vocal deviation for breathiness (seven mild, eight moderate and one severe), and seven showed asthenia (four mild and three moderate). In the postintervention period, all 17 individuals had an absence of grade of vocal deviation for breathiness (p = 0.0001) and for asthenia (p = 0.015). The Cronbach’s alpha value was very good, 0.88, indicating high reliability for the observers.

## DISCUSSION

Voice is one of the most striking characteristics of each person’s uniqueness, to the point that it can be used in forensic medicine ( 25 ). Voice is one of more important milestones of sexual dimorphism established at puberty (linked to GH and sex steroids), maintained in adulthood, and attenuated in senescence ( 26 , 27 ). The impact of severe IGHD on voice is so important that it counteracts the effect of puberty and aging on the structure of the vocal fold, thereby preventing the variation of its fundamental frequency associated with aging ( 13 ). This typical high-pitched voice helps establish, at any age, the diagnosis of IGHD just by listening. High-pitched voice is also found in subjects with a different mutation in the GHRHR gene ( 28 ) and in GH insensitivity syndrome ( 29 ). Accordingly, low-pitched voice is a common finding of acromegaly ( 30 ). Anecdotally, an opera singer with undetected acromegaly was noted to progressively change from tenor to baritone to bass, showing the effects of long-term exposure to GH and IGF-1 on voice ( 31 ).

These examples show the importance of the relationship between voice and GH. In different cultures, higher stature ( 26 ) and voice characteristics ( 32 ) are linked to higher social status, but the relevance of each factor to social status is presently unknown. In the present work, we used voice therapy and artistic intervention (choir singing) to test the hypothesis that voice training can affect quality of life. To this end, we took advantage of extended IGHD kindred with both severe short stature and a high-pitched voice ( 3 , 13 ). Their voice also has an accentuated grade of roughness, breathiness, and strain ( 12 ), and they exhibit low V-RQOL scores ( 13 ). Curiously, they rarely complain about their severe short stature, as evidenced by their normal general quality of life ( 15 ), while they often complain about their voices. These vocal complaints are not apparently related to stature, as they are very active professionally and sexually, often marrying normal-statured people, with affected or heterozygous offspring, and well accepted in the community.

GH replacement therapy (GHRT) during childhood is a natural way to improve stature and voice. Nevertheless, these IGHD individuals consider themselves ‘short long-lived people’, not patients. Therefore, after an initial curiosity for treatment, most of them (and their parents) became uninterested in a daily injectable GHRT ( 5 ). Additionally, they believe that they can live normally, with overall good functionality until advanced ages, a fact that was confirmed by our previous studies ( 4 , 5 , 11 ). In a previous study, we showed a positive effect of singing and voice therapy with SOVTE on the acoustic parameters of their voices depicted by a spectrogram ( 19 ). However, the impact of choir singing on the most relevant measures of social life, such as V-RQOL scores, vocal self-assessment, and auditory-perceptual analysis, the gold standard for characterizing vocal problems, was not evaluated. This assessment is the principal purpose of this work, as singing is often used to improve not only the voice but also, more importantly, general health ( 33 ).

In the current study, we used a validated and reliable Brazilian version of the V-RQOL, responsive to changes, which is useful in the evaluation of dysphonic patients ( 23 ). We observed an accentuated increase in total, physical and socioemotional V-RQOL scores, with three measures very close to 100 (the highest score), after the vocal intervention, with values that are comparable to healthy controls from the same region ( 13 ). These findings are similar in degree of improvement to what was observed in non-GHD patients with a similar warm-up protocol ( 18 ).

This increase in total, physical and socioemotional V-RQOL scores parallels the improvement in their vocal self-assessment. Before the vocal intervention, only one-third of them rated their voice as very good. After the intervention, almost 90% rated their voice as very good. Before the intervention, 65% of them rated their voice as fair, and after the intervention, less than 12% rated it as fair. Therefore, the vocal intervention had a remarkable effect on the subjective self-assessment of the voice.

The improvement in the self-assessed V-RQOL scores was accompanied by striking changes in the auditory-perceptual analysis (assessed by experienced and blinded judges). In the preintervention period, 7 IGHD individuals had a severe grade of general vocal deviation, and 10 had a moderate or severe grade of general vocal deviation. In the post-intervention period, all participants in the study had a reduction (or absence) in the grade general vocal deviation. All 17 IGHD individuals had some grade of deviation in roughness preintervention, while only 4 presented a mild deviation in postintervention assessment, indicating less vibratory irregularity ( 24 ).

Our data agree with others obtained in non-GHD dysphonic subjects, where the lower the roughness is, the higher the rate of improvement of general vocal quality with a vocal intervention ( 7 ). In the preintervention assessment, 16 of the 17 IGHD individuals had some grade of deviation in breathiness, and 7 had asthenia. After intervention, all 17 individuals had null grades of deviation in both parameters, with improvement of audible air signal and breathiness, probably due to better adduction of the vocal folds.

The improvement of asthenia may reflect muscle training. Preintervention, all 17 IGHD individuals had some grade of strain, while postintervention, only 6 presented a mild deviation, suggesting reduced phonatory effort during vocal production.

We have previously reported that these subjects presented with more strain, possibly necessary for self-assessment of voice, related to the observed higher prevalence of laryngeal constriction ( 12 ). We believe that the retroflex resonance, generated after blowing the tube in water in the SOVTE, favors reduction of the excessive effort of the vocal tract, improving the control and coordination of the breathing, and consequent reduction of the roughness and strain, as previously shown in non-GHD individuals ( 7 , 29 ).

These individuals appear to adjust their singing based on the ability to hear not only their own sound but also, the other voices of the choir. During singing, the choir functions as a superordinate system, or superorganism, that imposes boundary conditions on the dynamic features of the individual singers, a fact that is positively associated with well-being and quality of life ( 33 ). Accordingly, the current vocal improvements extended to conversational speech, thus favoring the social interaction of these subjects, possibly increasing well-being and self-confidence.

Short stature is usually associated with poor quality of life ( 34 ). Consequently, most people believe that being taller is a sign of higher status and social privilege. However, smaller bodies have numerous advantages in terms of health and longevity ( 35 ), shared by the Itabaianinha IGHD cohort ( 4 , 5 ). As these individuals seem to experience more positive than harmful consequences of IGHD, it seems reasonable not to give GHRTs to adult individuals. Additionally, GHRTs in adulthood may not have obvious benefits on their voice . Therefore, treating the harmful effects associated with IGHD, such as their high-pitched voice, with other measures, such as the use of SOVTE and choir singing, seems appropriate.

Our work has some limitations. First, we used a pooled group of both genders instead of separating by gender. However, the effect of IGHD seems to exceed that of sex, as was shown for *f0* , in which the effect of sex steroids was abolished ( 13 ). Second, we did not use a control group, and consequently, we cannot strictly compare the combined approach used in this work with other techniques in terms of cost benefits. However, this method suggests that the outcomes are impacted by the intervention (voice therapy and choir singing), since other possible elements of change did not occur at the same time as the intervention ( 21 ). It is extremely unlikely that 90 days of observation will have any effect on the variables studied, with the participants maintaining their normal routines. Third, we were not able to discern the muscular effect of SOVTE ( 7 , 18 ) from those of choir singing. We emphasize that our objective was not to observe separate effects of these techniques but rather to assess their overall impact on the social and auditory-perceptual aspects of the voice. However, we highlight that voice therapies are often complex and multidimensional in nature (and rarely offered in isolation). Therefore, the active ingredient underlying the effectiveness of most therapies is not well understood ( 36 ).

Art is very useful to bring science and the laboratory together. However, the task of “putting society in the laboratory” is a complex process. It requires transdisciplinarity in academia (often desired but not always achieved). In this sense, it is important to understand the difference between multidisciplinarity, interdisciplinarity, and transdisciplinarity. Multidisciplinary is based on knowledge of different disciplines but remains within its limits. Interdisciplinary analyzes, synthesizes, and harmonizes the links between disciplines into a coordinated and coherent whole. Transdisciplinarity, as noted in this article, integrates natural, social and health sciences in a humanities context and transcends their traditional borders. Art-science interaction requires the intersectoral cooperation between the academic sector and the private sector, civil society, and government ( 1 , 37 ). We hope that this work will contribute to: transdisciplinarity in academia and intersectoral cooperation with the government and civil society.

In conclusion, in adult IGHD individuals, an artistic intervention – choir singing and voice therapy with semioccluded vocal tract exercises – markedly improved V-RQOL scores, voice self-assessment, and auditory-perceptual analysis, thus improving their quality of life related to voice. This is particularly important in a setting where GH replacement therapy is not widely accepted.
